# Tapping doesn’t help: Synchronized self-motion and judgments of musical tempo

**DOI:** 10.3758/s13414-019-01722-7

**Published:** 2019-05-06

**Authors:** Justin London, Marc Thompson, Birgitta Burger, Molly Hildreth, Petri Toiviainen

**Affiliations:** 1 Department of Music, Carleton College, Northfield, MN 55057 USA; 2 University of Jyväskylä, Jyväskylä, Finland

**Keywords:** Music, Rhythm, Tempo illusion, Sensorimotor synchronization, Perception–action dissociation, Perceptual sharpening

## Abstract

For both musicians and music psychologists, beat rate (BPM) has often been regarded as a transparent measure of musical speed or tempo, yet recent research has shown that tempo is more than just BPM. In a previous study, London, Burger, Thompson, and Toiviainen (*Acta Psychologica, 164,* 70–80, 2016) presented participants with original as well as “time-stretched” versions of classic R&B songs; time stretching slows down or speeds up a recording without changing its pitch or timbre. In that study we discovered a tempo anchoring effect (TAE): Although relative tempo judgments (original vs. time-stretched versions of the same song) were correct, they were at odds with BPM rates of each stimulus. As previous studies have shown that synchronous movement enhances rhythm perception, we hypothesized that tapping along to the beat of these songs would reduce or eliminate the TAE and increase the salience of the beat rate of each stimulus. In the current study participants were presented with the London et al. (*Acta Psychologica, 164,* 70–80, 2016) stimuli in nonmovement and movement conditions. We found that although participants were able to make BPM-based tempo judgments of generic drumming patterns, and were able to tap along to the R&B stimuli at the correct beat rates, the TAE persisted in both movement and nonmovement conditions. Thus, contrary to our hypothesis that movement would reduce or eliminate the TAE, we found a disjunction between correctly synchronized motor behavior and tempo judgment. The implications of the tapping–TAE dissociation in the broader context of tempo and rhythm perception are discussed, and further approaches to studying the TAE–tapping dissociation are suggested.

## Introduction: Rhythm, movement, beats, and tempo

In perhaps no other domain are perception and action so intertwined as in musical rhythm. As Chen, Penhune, and Zatorre (2008) aptly put it, “listening to musical rhythms recruits motor regions of the brain”; they found the supplementary motor area (SMA), midpremotor cortex (PMC), and cerebellum to be activated when listening to musical rhythms, whether or not motor planning or actual activity was involved. Dalla Bella, Bialunska, and Sowiński (2013) posit this is due to music’s “peculiar and regular beat and metrical structure” (p. 14), which is lacking in other periodic or quasiperiodic stimuli, such as speech. Ross, Iversen, and Balasubramaniam (2016) survey studies on the role of the motor system in predictive rhythmic movement and present evidence for a causal role of motor planning and simulation in rhythm perception. The links between rhythm perception and production provide the impetus for common coding theories/models of rhythm perception and production (Maes, Leman, Palmer, & Wanderley, 2014; Ross et al., 2016; see also Knoblich & Flach, 2001).

Rhythmic structure is encoded in our bodily movements as we listen to music, and movement enhances our rhythmic awareness. Toiviainen, Luck, and Thompson (2010) showed how different metrical levels are mapped onto different parts of the body (see also Leman, 2008). Su and Pöppel (2012) found that bodily movement assisted in pulse finding and improved participants’ synchronization with an imperfect metronome, and Manning and Schutz (2016) found that movement enhances sensitivity to rhythmic perturbation for both musicians (especially percussionists) and nonmusicians. Moreover, Manning and Schutz found that when participants were not allowed to move, the percussionists fared no better than nonpercussionists in a temporal judgment task.

It is not surprising that musicians—who, by definition, have spent long hours practicing precisely timed bodily movements in making music, often in synchrony with other musicians—fare better than nonmusicians at many rhythmic synchronization and judgment tasks, and that percussionists are better than other musicians, given that tapping along with a rhythm or melody is the most typical synchronization task/measure (Cameron & Grahn, 2014; Manning & Schutz, 2016; Repp, 2005; Repp & Su, 2013; Slater & Kraus, 2015). Musicians are able to synchronize more accurately over a wider range of tempos than are nonmusicians (Scheurich, Zamm, & Palmer, 2018), and Hurley, Martens, and Janata (2014) found that musically trained participants were more likely to tap along with music than untrained participants. When tapping along with a simple metronomic stimulus, musicians and nonmusicians differ in terms of the tendency to tap slightly ahead of the metronome, the so-called negative mean asynchrony, as it is smaller in musicians than in nonmusicians (see Aschersleben, 2002; Scheurich et al., 2018).

**Fig. 1:**
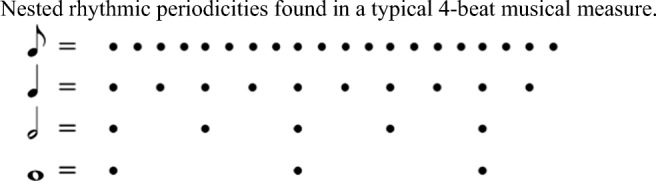
Nested rhythmic periodicities found in a typical four-beat musical measure

Musical melodies and rhythms are of course more complex than metronomes, and one aspect of this complexity is that musical melodies and rhythms typically involve multiple, related periodicities. For example, a melody composed of a stream of eighth notes in 4/4 time, where each note was 200 ms in duration, could give rise to periodic structures (what a musician would call rhythmic or metrical layers) at 200-ms, 400-ms, 800-ms, and 1,600-ms intervals (see Fig. 1).

Such rhythms afford more than synchronization rate—the hypothetical melody just given would allow for hearing either a relatively brisk 400-ms periodicity (the quarter note level) or a more leisurely 800-ms periodicity (the half-note level). Martens (2011) asked participants to tap along with music that afforded both faster and slower synchronization rates and found three distinct synchronization strategies: (a) slow tappers, who consistently tapped at slower periods; (b) fast tappers, who consistently tapped at faster periods; and (c) “switchers,” who tapped at both fast and slow periods—though “switchers” did not change their tapping rate within any given trial. Whether one is a “fast tapper” or a “slow tapper” can be related to one’s spontaneous motor tempo (SMT), also known as personal tempo or natural pace. SMT is measured by asking participants, absent any external rhythmic stimulus or context, to simply tap or walk at a comfortable rate that is neither too slow nor too fast. Although most adults show SMTs in a range of 500 ms to 600 ms (Fraisse, 1982; McAuley, 2010), it can range from 300 ms to 800 ms, and it changes over one’s life span (McAuley, Jones, Holub, Johnston, & Miller, 2006). These same preferred tempos are the most common periodicities found in a wide range of music (McKinney & Moelants, 2006; Parncutt, 1994; van Noorden & Moelants, 1999). SMT has been shown to have a bearing on rhythm task performance (McAuley, 2010; Repp, 2005), and its development can be affected by musical training (Scheurich et al., 2018).

When we hear a melody or rhythm, we typically have a clear sense of its speed or tempo. Indeed, our ability to discriminate tempo differences—that is, a change in period for a series of tones or drumbeats—is quite sensitive. It is far more acute than our ability to discriminate the duration of isolated temporal intervals, with just-noticeable differences (JNDs) for tempo changes ranging from 1% to 3%, versus 6% for isolated intervals (Drake & Botte, 1993; McAuley & Kidd, 1998; Miller & McAuley, 2005). Likewise, our memory of the tempo of previously heard performance or recording can be quite precise. Levitin and Cook (1996) found that memories for the tempo of recorded songs was near to a JND for tempo discrimination, and argued that our memory for tempo is absolute. Jakubowski, Farrugia, Halpern, Sankarpandi, and Stewart (2015), in a study of “earworms,” found that tempo ratings for involuntary musical memories were again highly accurate, though Moelants, Styns, and Leman (2006) found weaker evidence for absolute tempo memory. Lapidaki (2000) asked a group of musicians and nonmusicians to optimize the tempo of several pieces of classical and popular music using the method of adjustment and found that some musician participants were extremely consistent in their adjustments on repeated trials, though overall this finding was not statistically significant. Gratton, Brandimonte, and Bruno (2016) had musicians and nonmusicians perform both tempo labeling and tempo reproduction tasks that involved seven distinct tempo levels across a wide range. They found that musicians were better at tempo identification than nonmusicians were, but no difference between the groups in the tempo-reproduction task.

For both musicians and music psychologists, beat rate (beats per minute, or BPM; often marked in musical scores as “MM” for “Mälzel’s Metronome”) is often regarded as a transparent measure of musical speed (Jones & Boltz, 1989; Moelants et al., 2006; Parncutt, 1994; Quinn & Watt, 2006; van Noorden & Moelants, 1999). *Beat* itself is a somewhat ambiguous term. Intuitively, we think of it as the rate at which we tap our toe or otherwise move along with the music, and while it tends to be the periodicity closest to the 500-ms to 600-ms range (i.e., congruent with average SMTs), some rhythms and melodies afford more than one possible beat rate, as noted above. Moreover, BPM is not the only cue for tempo, as other factors such as loudness, register (high vs. low frequency range), melodic contour, and timbre/tone color can affect tempo judgments (Boltz, 2011; Drake, Gros, & Penel, 1999; London, 2011). Nonetheless, BPM is still regarded as the dominant cue for musical tempo, given its role in grounding the temporal framework for rhythmic perception and action.

In a previous study that explored tempo perception and observed movement (London, Burger, Thompson, & Toiviainen, 2016), we obtained an unexpected result. That experiment used a number of classic Motown songs as stimuli, specifically chosen for their high degree of beat clarity and sense of “groove” (Janata, Tomic, & Haberman, 2012), both of which we presumed would make their tempos relatively clear. Stimuli were also matched so that other cues for tempo were kept relatively constant, insofar as is possible with real as opposed to artificial musical stimuli. In that study, our main interest was to see the effect of an ecologically valid visual cue (a motion-capture-generated dancing figure) presented with the correspondingly ecologically valid musical stimuli; our main finding was that vigorous movement led to judgments of faster tempo in contrast to the audio stimuli presented alone—hence, we presented participants with both audio-only and audio-plus-video stimuli. In our 2016 study, the participants’ task was to rate the tempos of individual stimuli using a 7-point Likert-type scale (see Method section below for further details of the stimuli and experimental task, which are the same in the current experiment).

To forestall reflexive associations between a particular song and a particular tempo rating, in our previous experiment each stimulus was presented at both its original tempo as well as in “time-stretched” versions. Time stretching speeds up or slows down the tempo of an audio file while keeping its pitch/frequency stable; moderate degrees of time stretching also preserve the timbre without introducing audible artifacts of the digital signal processing. Stimuli were presented at their original tempos of 105, 115, and 130 BPM, as well as in time-stretched variants (±5%). This yielded eight different tempos from 100 to 135 BPM, each separated by approximately five BPM. These five-BPM increments are well above a JND for tempo discrimination in a beat-based context (Drake & Botte, 1993; Miller & McAuley, 2005), and the stimuli were in a range where tempo and rhythmic discrimination has been shown to be most acute (Fraisse, 1984; Grondin, 2010; Madison & Paulin, 2010; Repp, 2005).

Given that the tempo cues in these stimuli were robust and unambiguous, in the audio-only condition we expected that participants would (a) be able to sort out the tempos of the original and their time-stretched versions of all of the stimuli, and (b) that their ratings would correspond to the BPM rates of the stimuli, as we had endeavored in our selection of stimuli to keep other tempo cues constant. Although they could sort out the tempos of the original and time-stretched stimuli without difficulty, our participants’ ratings did not correspond to the BPM rates of the stimuli. Rather, our participants consistently inflated the ratings of sped-up versions of a song and correspondingly deflated the ratings of slowed-down versions. For example, the average ratings of sped-up songs from the slowest tempo group (originally 105 BPM, now 110 BPM) were rated as faster than average ratings of songs from the middle tempo group at their original tempo level (115 BPM). To put it another way, in the context of this experiment and rating task, BPM was longer the dominant cue for tempo ratings. We described this tempo rating error as a song-specific tempo anchoring effect (henceforth TAE), as the perceived tempo of each song is “anchored” around the original version.

Here we report on an experiment that investigated the effect of overt movement on the TAE. Given the strong links between motor behavior and rhythm perception and production as noted in the literature summarized above, our hypothesis was that tapping to the beat of the music—an overt motor behavior—would give participants a greater sense of the music’s BPM rate and thus make the beat rate a more salient cue for tempo judgments, which in turn should reduce or eliminate the TAE. As we introduced a movement component to the participants’ responses, we also collected data on participants’ SMTs (pretrial and posttrial) to assess if their tapping behavior and/or tempo judgments were affected by their “default” rate of movement.

## Method and apparatus

In this experiment, the stimuli from London et al. (2016) were presented to participants in two conditions: a nonmovement condition, as in the original experiment, and a movement condition, where participants tapped in synchrony to each stimulus. SMT data were collected pretest and posttest, and in the nonmovement condition the ability of participants to correctly rate the tempo of a drum pattern (i.e., an unambiguous beat-based tempo cue) was also assessed.

### Participants

Twenty-one participants (13 female) were recruited from the Carleton and Northfield, MN, community for the experiment. The average age was 22.8 years (*SD* = 7.9 years, mostly due to two older participants, 37 and 53 years); all other participants were between ages of 18and 23 years. Six participants had more than 10 years of musical training; eight had 5 to 10 years of training; and seven had less than 5 years of training. Ten participants were familiar with five or six of the songs used as stimuli in the experiment, five with three to five songs, and six participants were familiar with two or fewer songs. Participants were not directly compensated, but instead were entered into a drawing for a gift card to a local coffee shop.

### Stimuli

The sources of the audio stimuli used in the experiment are shown in Table 1.

**Table 1:** Musical stimuli used in the experiments

Artist	Title	Original BPM	R&B chart ranking	Sub-band flux
Temptations	“Get Ready”	134.5	#1 (1966)	492.02
Supremes	“Where Did Our Love Go?”	133	#1 (1964)	269.11
Supremes	“Stop, In the Name of Love”	117	#2 (1964)	474.34
Wilson Pickett	“The Midnight Hour”	113	#1 (1965)	397.57
Stevie Wonder	“Signed, Sealed, Delivered”	105.5	#1 (1970)	479.30
Temptations	“My Girl”	103	#1 (1964)	409.54

BPM = beats per minute; R&B = rhythm and blues

Although BPM is the dominant cue for tempo, other factors—such as event density, meter, register, timbre, and dynamics—can also influence tempo judgments (Boltz, 2011; Drake et al., 1999; Elowsson & Friberg, 2013; London, 2011). To control for these secondary factors, a homogenous set of musical stimuli were carefully chosen for the experiment. All stimuli are in simple duple meter (i.e., 4/4) with a light amount of “swing” (i.e., no overt triplet subdivision), and all are well established classics of the American 1960’s R&B style. At each core tempo level a pair of stimuli were chosen that balanced high versus low surface density. A score-based analysis of each song was performed that indexed the number of notes at the eighth-note level of the meter (i.e., binary division of the tactus) in each bar of the vocal melody, bass, and percussion parts. From these measurements, aggregate rhythmic density scores were calculated for each song, to ensure matched pairs at each tempo level. As a corresponding measure, the low-frequency spectral flux for each song was calculated by choosing an octave-wide frequency range between 100 Hz and 200 Hz and calculating the sub-band flux (MIRtoolbox function “mirflux”) by taking the Euclidean distances of the spectra for each two consecutive frames of the signal (Alluri & Toiviainen, 2010), using a frame length of 25 ms and an overlap of 50% between successive frames, and then averaging the resulting time series of flux values.

Two independent raters who tapped along to each song using a beat-finding metronome produced BPM rates that were averaged, and their rates were also checked using the MATLAB-based MIRtoolbox “mirtempo” function (Lartillot & Toiviainen, 2007). The original versions were first time-stretched so their tactus rates exactly aligned at 105, 115, or 130 BPM using Audacity (Version 2.0.5), an open-source sound editor (audacity.sourceforge.net). The stimuli were then time-stretched a second time to produce additional stimuli that were ±5% of these three baseline rates, yielding a total of 18 stimuli. The BPM rates of the time-stretched stimuli were then rechecked by the independent raters and the “mirtempo” beat-tracking algorithm, and all confirmed the stimuli were at the correct BPM rates (see Table 3). These core tempos and time-stretch amounts were intentionally chosen to produce a set of stimuli that incrementally spanned the 100–135 BPM range. All stimuli were 10 seconds in duration, and each began on the first significant downbeat following the introductory portion of each song.

In addition to the musical excerpts, in the nonmovement condition, 10-second clips of a standard rock drum pattern (the same as used in the pretest) were interleaved with the song stimuli. Rock drum patterns were at 100–135 BPM in five BPM increments (hence eight additional stimuli). These were included as a check to see if participants could make veridical judgments of a generic rhythmic stimulus, as well as to assess the scale used for the tempo judgments to determine if the TAE was perhaps an artifact of a compressed rating scale. Pilot trials had shown that participants were able to give accurate ratings for the drum patterns in the nonmovement condition (as was also borne out in the main trials), and so they were not included in the movement condition, as synchronized movement would not provide any additional benefit.

### Apparatus and procedure

**Fig. 2:**
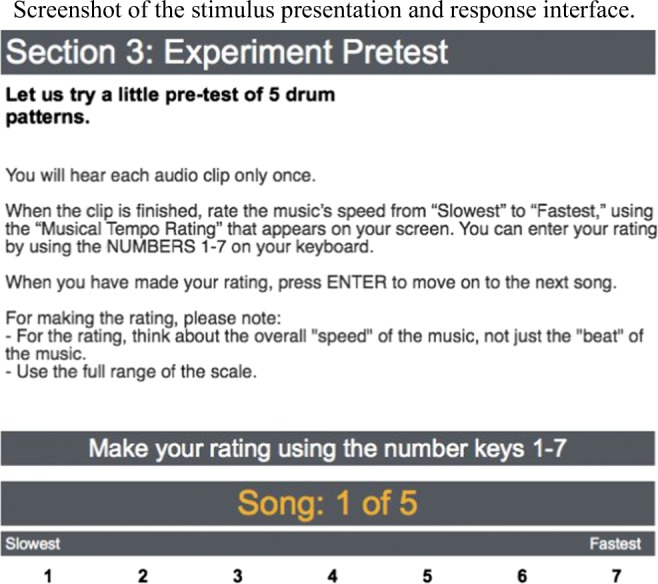
Screenshot of the stimulus presentation and response interface

The experimental task in each trial involved making an absolute tempo rating of each stimulus using a 7-point Likert-type scale. Figure 2 is a screenshot of the stimulus presentation and response interface used in both the pretest and the main trials. Note that on every trial participants are reminded to focus on the overall speed of each stimulus (rather than simply its BPM rate), to use the full range of the 7-point scale, and that 1 = *slow* and 7 = *fast.*

Trials were presented in two blocks (counterbalanced orders): movement versus nonmovement. Participants did one block of trials on a given day, and then returned 1–3 days later to do the other block. Stimuli were presented in a unique random order for each participant within each block, with the randomization constrained so that different versions of the same stimulus were not presented consecutively. Stimuli were presented to participants in quiet rooms on a MacBook Pro laptop computer (13-inch screen size, 2.65 GHz Intel Core i5, with 3 or 4GB RAM, running OS 10.10.3), via a custom-made interface in Max, a graphical programming environment (Version 7.3.5; www.cycling74.com). Participants listened via Sennheiser HD280 Pro headphones, which provided additional attenuation of ambient noise, with the headphone volume adjusted to a comfortable listening level. Each block took 20–25 minutes to administer.

An introduction and pretest were administered prior to both experimental sessions. The introduction presented songs at the low and high ends of the tempo range, as well as time-stretched versions of a Motown R&B song to familiarize participants with the types of stimuli used in the experiment (demo songs were not used in the experiment). Knowing that they would hear various time-stretched versions of a song, participants realized that each time they heard a particular song they would need to make a fresh tempo judgment. The pretest then presented participants with a simple rock drumming pattern (kick drum, snare, and hi-hat sounds) to indicate the range of tempos used in the experiment (100–135 BPM) as well as to familiarize them with the response interface and tempo rating procedure.

In the movement/tapping condition, during the pretest, participants tapped at the beat rate with the drumming patterns in the pretest as well as with the songs in the main trials. To gather participant SMT data in the tapping experimental block, both before the pretest and after the main trials participants were asked to tap on the space bar of the computer “at a comfortable rate that is neither too fast nor too slow”; two 25-second tapping runs were collected.^1^ Participant taps generated a high-pitched percussion sound (a woodblock), which was heard along with the stimulus; previous studies in sensorimotor synchronization have shown that such auditory feedback—essentially, a clear effect of the motor action that is not confused with the pacing stimulus—enhances synchronization accuracy (Repp, 2005; Repp & Su, 2013). In the pretest and main trials, participants were instructed to start tapping as soon as they had a clear sense of the beat (usually within 1 to 2 seconds from the start of the stimulus), and had to tap to the end of the entire clip before they were able to enter a tempo judgment.

For both tapping and nontapping blocks, a random delay of 4–6 seconds (during which time participants heard random environmental sounds—surf, seagulls, etc.) was inserted between trials to forestall carryover effects from trial to trial. The participant would then cue the next stimulus. Lastly, data on each participant’s musical background were collected using a computer-based form.

## Results

No effects due to musical training or stimulus familiarity were found. Regarding training, a Mixed ANOVA, with condition (tapping vs. nontapping), core tempo (three levels), and stretch (three levels) as within-participant variables, and musical training (<5 years, *n* = 7, vs. >5 years, *n* = 14) as a between-participant variable, found no main effect of training, *F*(1, 19) = .256, *p* = .610, nor were there any significant two-way interactions between training and the within-participant variables. We ran a similar mixed ANOVA, with familiarity as the between-participant variable (familiar with 0–2 songs, *n* = 7, vs. familiar with 3–6 songs, *n* = 14) again and found no main effect of familiarity, *F*(1, 19) = .141, *p* = .711, and no significant two-way interactions.

### Tempo-rating data

#### Song stimuli

**Fig. 3:**
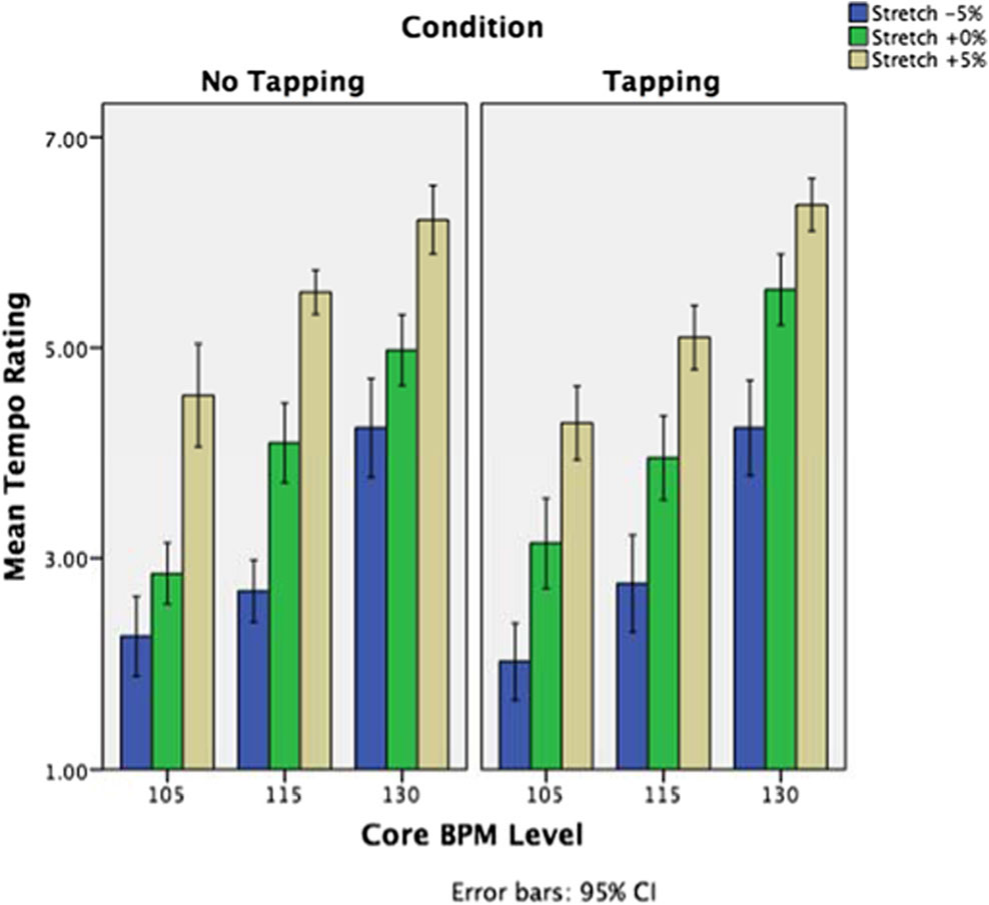
Average participant tempo ratings (*y*-axis) for stimuli, grouped by core tempo levels. Left panel no tapping condition; right panel tapping condition. Error bars indicate 95% confidence interval

A 3 × 3 × 2 repeated-measures ANOVA (Core BPM × Stretch × Movement Condition (tapping vs. no tapping) as the independent variables, and tempo rating as the dependent variable) found main effects for BPM, *F*(2, 40) = 149.270; *p* < .001, η_p_^2^ = .882, and stretch, *F*(2, 40) = 233.205, *p* < .001, η_p_^2^ = .921, but none for movement condition (see Fig 3). The interaction between BPM and movement condition was significant, *F*(1.496, 29.930) = 4.134, *p* = .023, η_p_^2^ = .171, Greenhouse–Geisser correction applied, with mean tempo ratings being slightly lower at 105 and 115 BPM in the no tapping condition, but slightly higher at 130 BPM. The interaction between movement condition and stretch was significant, *F*(2, 40) = 4.057, *p* = .025, η_p_^2^ = .169, as small differences between −5% and 0% stretch at 105 BPM and 130 BPM in the no tapping condition became more pronounced in the tapping trials (see Fig 3). Lastly, there was an interaction between core BPM and stretch, *F*(2, 40) = 3.086, *p* = .020, η_p_^2^ = .124, again having to do with the small difference between −5% and 0% stretch at the 105 BPM level.

As can be seen in Fig 3, the TAE was replicated in both the tapping and no tapping trials. Post hoc pairwise *t* tests (Bonferroni correction applied for multiple comparisons) found statistically significant differences in tempo ratings between all adjacent BPM levels (*p* < .001, save for 105 BPM −5% vs. 105 BPM 0% in the no tapping condition (*p* = .009), and 115 BPM +5% vs. 130 BPM −5% in the tapping condition (*p* = .003). Where stimuli in each panel of Fig. 3 appear to have similar ratings, the differences are mostly nonsignificant (i.e., their ratings effectively are the same), save for no tapping at 105 BPM + 5% versus no tapping at 115 BPM + 0%,  *p* = .043.

Table 2 gives the grand averages of the tempo ratings for each core BPM level in both tapping and no tapping conditions.

**Table 2:** Grand average of tempo ratings for each core BPM category, no tapping (NT) and tapping (T) conditions

NT rating	T rating	Core BPM
3.22	3.15	105
4.10	3.94	115
5.14	5.38	130

BPM = beats per minute

Differences between no tapping and tapping conditions were not significant. As one would expect from Table 2, bivariate correlations between grand mean of the ratings for each core tempo level and their corresponding BPM rates were very robust in both no tapping and tapping conditions (*r* = .998, *p = .*043 and *r* = .999, *p* = .034, respectively).

### Rock drum patterns

**Fig. 4:**
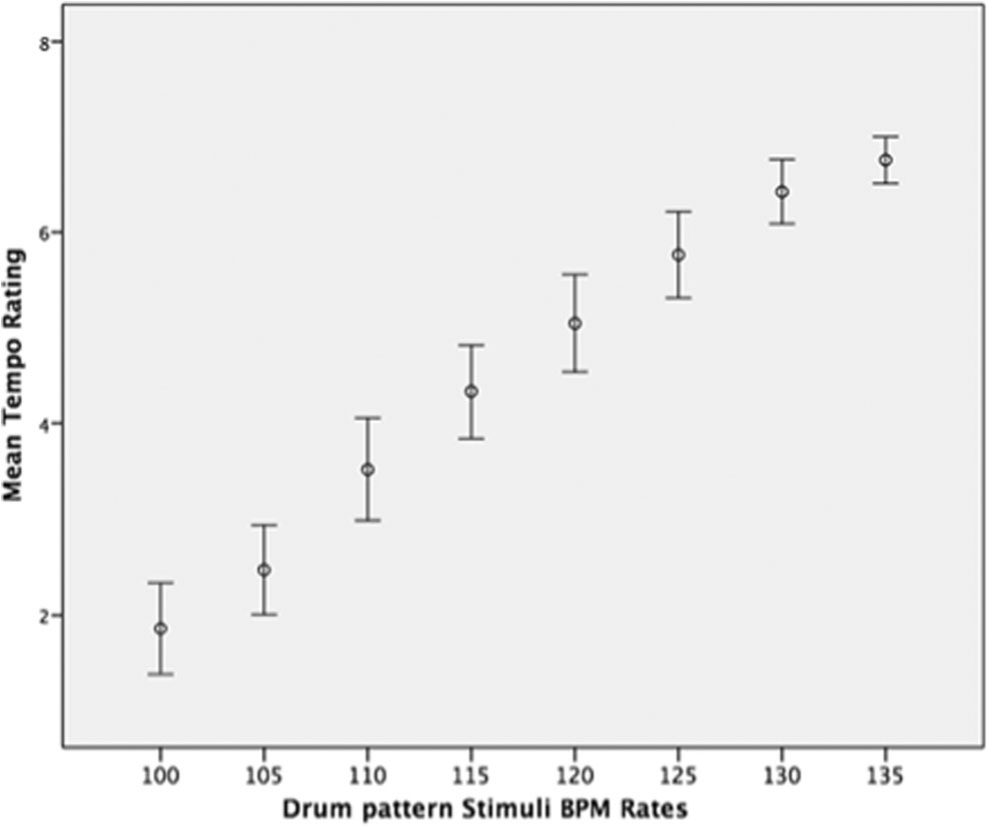
Average tempo ratings of rock drumming stimuli (Experiment 2, no tapping condition). Error bars indicate 95% confidence intervals. BPM = beats per minute

Participant tempo ratings for the rock drumming stimuli interleaved among the song stimuli are given in Fig. 4.

As one would expect from Fig. 4, a repeated-measures ANOVA found a significant effect for BPM rate, *F*(7, 140) = 80.569, *p* < .001, η_p_^2^ = .801. Post hoc pairwise comparisons (Bonferroni correction applied) were nonsignificant for adjacent BPM rates, save for 105 and 110 BPM (*p* = .010). All other pairwise comparisons were highly significant (*p* < .005, save for 115 and 125 BPM, *p* = .016, and 120 and 130 BPM, *p* = .012). Even though the rock drum pattern stimuli appeared within the context of rating original and time-stretched versions of real songs, participants had no trouble producing absolute tempo ratings that corresponded to the BPM rates of the drum stimuli (*r* = .994, *p* < .001). This is in contrast to their ratings of the R&B song stimuli, which did not.

### Tapping data

#### Main trials tapping data

As tapping did not appear to affect the TAE, participants’ tapping data were analyzed to ensure that they were actually tapping in synchrony (that is, at the same beat rate) with the stimuli. As noted by Martens (2005, 2011), participants can synchronize using different tapping rates for the same stimulus—either tapping every beat (“fast tappers”) or every other beat (“slow tappers”). Five of the 21 participants were slow tappers, tapping at twice the beat rate in six or more trials (>33% of all trials); 11 participants were slow tappers in at least one trial. Slow tapping appeared at all core BPM rates, though slow tapping was more likely at slower core BPM rates (at the 130 BPM core tempo there were 20 slow tapping trials, at 115 BPM there were 24, and at 105 BPM there were 38). This was surprising, as one would normally expect a bias toward slow taps at the fastest rather than the slowest BPM rate, keeping one’s tapping rate as close to 2 Hz as possible (see Discussion for further comment).

Having identified the five slow tappers, we reran the 3 × 3 × 2 (Core BPM × Stretch × Movement Condition) mixed-model ANOVA, with tapping mode (slow vs. fast) as a between-groups variable. We found (unsurprisingly) main effects for BPM, *F*(2, 38) = 114.923, *p* < .001, η_p_^2^ = .858; stretch, *F*(2, 38) = 195.774, *p* < .001, η_p_^2^ = .912; but no main effect of movement condition (tapping vs. no tapping), *F*(1, 19) = .913, *p* = .351, or, most notably tapping mode (slow vs. fast), *F*(1, 19) = .254, *p* = .620. There were near-significant interactions between BPM and movement condition, *F*(2, 38) = 2.993, *p* = .062, and movement condition and tapping mode, *F*(4, 76) = 3.328, *p* = .084.

Table 3 gives the objective beat rate at each time-stretch level (calculated from MIR toolbox and checked by independent tapping ratings of two experimenters using a MIDI drum pad), the grand average participant tapping rate, the standard deviation of the average tapping rates, and the difference between objective and average tapping rates. The tapping rates reported in Table 3 for slow tappers were corrected by dividing their tapping intervals in half. Note that as time-stretched versions were created by increasing or decreasing the tempo by 5% from the core rates at 130, 115, and 105 BPM, the BPM intervals at the two 110 BPM columns do not precisely match.

**Table 3:** Tapping data, averaged across all participants

BPM categories	135	130	125	120	115	110	110	105	100
BPM interval	438	462	485	496	522	548	543	570	600
Mean tap interval	443	462	486	496	516	545	541	560	591
*SD* of tap interval	43	41	43	47	55	44	60	65	54
Mean BPM vs. tap	−5	0	−1	0	6	4	2	10	9

All measurements in ms, corrected for “slow” tappers. BPM = beats per minute

The data make clear that at all tempos participants were tapping at 1 times or one-half times the beat rate, as evidenced by the extremely small differences between the “objective” BPM intervals and the mean intertap intervals. A repeated-measures ANOVA, with the grand averages of participants’ tapping rates for each BPM category as the independent variable (nine levels) found (unsurprisingly) a main effect for tapping rate, *F*(2.583, 105.912) = 195.328, *p* < .001, η_p_^2^ = .827, Greenhouse–Geisser correction applied; post hoc pairwise comparisons (Bonferroni correction applied) found tapping rates between adjacent BPM categories to be highly significant (*p* < .001), save for the two categories at 110 BPM (more precisely, 548-ms and 543-ms periodicities), *ns*, and between 105 and 100 BPM (*ns*). This is in accord with a general tendency toward greater tapping variability as the stimulus tempo decreases/intertap interval increases (Repp, 2005). The standard deviation of intertap intervals was 8%–11.6% (average 9.7%) of the mean intertap interval, in accordance with Weber’s law.

#### Pretest–posttest spontaneous motor tempo

As a final check on participant reliability, we examined the spontaneous motor tempo (SMT) data gathered in the pretest and posttests; data are summarized in Table 4. A statistically significant increase in mean SMT from pretest (677 ms) to posttest (612 ms) was evident, *t*(20) = 2.916, *p* = .009. Although the increase in mean and median tapping intervals may be partially an arousal effect, most likely this is a carryover effect from the experiment, as most participants’ posttest spontaneous tapping rate moved toward the mean BPM interval for all stimuli used in the experiment (510 ms); in the pretest, five participants tapped at a rate within ±50 ms of the 510 ms grand mean of the stimuli, whereas posttest, 12 participants were within ±50 ms of 510 ms. Participants’ pretest versus posttest SMTs were strongly correlated (*r* = .791, *p* < .001).

**Table 4:** Pretest and posttest mean spontaneous tapping rates, in milliseconds

Measure	All pretest	All posttest	Fast pretest	Fast posttest
Mean tap interval	677	612	641	573
Median tap interval	637	540	605	536
*SD* of mean	145	152	110	91
Minimum tap interval	495	486	534	486
Maximum tap interval	1,023	1,019	887	816

A wide range of participant SMTs was also evident; moreover, there was no clear clustering of tapping rates. A between-subjects ANOVA found no statistically significant difference between the mean SMTs of participants with less than 5 years of musical training versus those with 5 or more years of training in either pretest or posttest—pretest, *F*(1, 20) *=* .513, *p* = .483; posttest *F*(1, 20) *=* 1.547, *p* = .229. A point-biserial correlation found no correlation between tapping behavior in the main trials (slow vs. fast) and pretest tapping rate (*r*_pb_ = .351, *p* = .119); when only the “fast tappers” are examined (i.e., slow tappers excluded), mean and median pretest and post-test SMTs are similar to the overall rate, though as one would expect lower variability in their tapping is evident (see Table 4, right-hand columns). Indeed, pretest SMTs for all participants did not significantly correlate with the grand average of their within-experiment tapping rates (*r* = .219, *p* = .340).

In sum, all participants exhibited normative accuracy and typical rates in a spontaneous motor tapping task. While some were slow or fast tappers (and perhaps a few “switchers,” as per Martens, 2005, 2011), during the main trials, all participants’ tapping behaviors indicated an accurate sense of the beat rate of each stimulus, though their tempo rating data did not.

## Discussion

The tempo anchoring effect (TAE) has been described by London et al. (2016) as a conflict between absolute versus relative judgments of tempo that arises when listeners are presented with time-stretched versions of real music. Although they are able to correctly assign tempo ratings to faster versus slower versions of the same song, their absolute tempo judgments become nonveridical in that they no longer directly correspond to the beat rate of the stimulus. The current experiment examined the effect of focused motor engagement on the TAE by having participants make tempo judgments in both movement and nonmovement conditions. In the movement condition, participants tapped along to the beat of each stimulus before making their tempo judgments. Our hypothesis was that overt movement would reduce or eliminate the TAE by making the beat rate of each stimulus more salient. Our data do not support this hypothesis, as tapping along had no effect on the TAE.

In our study, pretest and posttest SMT data were collected in order to examine any correlation between SMT and tapping behavior and/or tempo judgments, but essentially we found none, as SMT data from the pretest did not predict tapping behavior in the experimental trials, nor was it correlated with tempo judgments (i.e., participants with slower SMTs did not tend to make slower overall tempo judgments). Previous studies have suggested that whenever possible, we try to keep periodic behaviors near a rate of 120 BPM/500 ms (i.e., 2 Hz), as it seems to be an optimal rate for movement and synchronization (Styns, van Noorden, Moelants, & Leman, 2007; van Noorden & Moelants, 1999). Yet, in our experiment, the opposite tendency was observed: As BPM rates decreased (i.e., the music got slower), the likelihood of slow tapping increased, making these periodic behaviors as far as possible from the optimal rate; at the slowest tempo, double-beat tapping periods were at the rate of 50 BPM/1.2 seconds/.833 Hz. Rather, the slower perceived tempos of the slower songs induced some participants to move relatively slowly along with them. Likewise, we found no effect of musical training on the TAE or acuity of tempo judgments more generally (contra Gratton et al., 2016; Manning & Schutz, 2016), nor was there any effect of familiarity—even participants who only knew one or two of the songs used in the experiment exhibited the TAE to the same degree as those who knew all or almost all of the songs.

In the current experiment, generic drum patterns were included among the nonmovement stimuli as a check on our participants’ ability to make absolute tempo judgments. Their tempo ratings of the drum patterns corresponded strongly to their actual BPM rates; when called upon to make an absolute tempo judgment of a generic rhythmic stimulus, they had no difficulty doing so. Participants were able to make these absolute tempo judgments of the drum stimuli even though they were interleaved with the time-stretched musical stimuli. Although the participants’ ratings for the drum stimuli make it clear that the 7-point scale used in the experiment was fine grained enough to individuate the BPM rates of our stimuli, it is also clear from both the drum and song data that there is some compression of the ratings toward the middle values of the 7-point scale. As one might expect with an absolute rating task, participants avoided the extreme values of the scale, except for the fastest and slowest stimuli. This, in turn, pushed the ratings of the remaining stimuli closer together, and this compression puts the absolute versus relative rating tasks in conflict—and we acknowledge that this contributes to the ratings overlap that is characteristic of the TAE. Thus, one might argue that the TAE is simply an artifact of the experimental design—specifically, the use of time-stretched stimuli in different tempo subranges of a limited rating scale. However, there are several reasons why the TAE may be more than this.

The first is the extent of the TAE. For example, if one gives the unstretched stimuli for the set of stimuli centered on 105 BPM—the slowest set of stimuli—a rating of “3” (rather than a “2”), and then differentiate the time-stretched versions accordingly, one might then expect a modest overlap (e.g., rating a 105% + 5% stimulus as a “4”, which would have been at the same tempo rating for the unstretched stimuli at 115 BPM). Our participant ratings, however, went beyond this, exaggerating the extent of the overlap (see Table 5). Second, we also note that stimuli were presented in unique random orders for each participant. For it was not the case that the core tempo versions of stimuli (i.e., those with no time stretch) were always heard first, and then served as a standard for subsequent stimuli. It was just as likely a participant heard the fastest or slowest version first, and then made her or his ratings of the other versions relative to it. Finally, it may be that time-stretching introduces subtle melodic or timbral distortions that, while not obvious digital processing artifacts, nonetheless serve as cues of time-stretching, and induce a heightened sense of tempo change. Thus in addition to a task/context-related shift of criterion, we speculate as to whether the TAE might also be an illusory tempo shift—that is, one that exceeds the “veridical” tempo change as measured by beat rate alone.

**Table 5:** Grand average of tempo ratings for all stimulus categories, no tapping (NT) and tapping (T) conditions

NT rating	T rating	BPM
2.26	2.02	−5%
2.86	3.14	105
4.55	4.29	5%
2.70	2.76	−5%
4.10	3.95	115
5.52	5.10	5%
4.24	4.24	−5%
4.98	5.55	130
6.21	6.36	5%

BPM = beats per minute

What the TAE does make clear is that beat rate is not always the most salient cue for musical speed, even though our stimuli were specifically chosen for their high degree of “groove” (i.e., high beat salience and affordance for pleasurable motor engagement; see Janata et al., 2012). Nor did synchronized movement seem to make the beat rate more salient. Although Su and Pöppel (2012) and Manning and Schutz (2016) found that movement increases sensitivity to local rhythmic disturbances, and although various studies have shown that movement can enhance beat tracking and/or disambiguate meter (Chemin, Mouraux, & Nozaradan, 2014; Drake et al., 1999; Maes et al., 2014), in our experiment, overt movement that was synchronized to the BPM rate (or one-half the BPM rate for “slow” tappers) of each stimulus did not reduce or eliminate the TAE.

Thus, we found a dissociation between rhythmic perception and action: Having established that the tempo perception of a stimulus is nonveridical, we found an associated motor action requiring the perception of event rate that is nonetheless highly accurate. Musical perception-action dissociations have previously been found in individuals that have a perceptual deficit, specifically pitch perception versus production for tone-deaf individuals (Loui, et al. 2008) and rhythm perception versus production for so-called beat-deaf individuals (Phillips-Silver et al., 2011; Sowiński & Dalla Bella, 2013), in which individuals cannot synchronize to a musical beat, but are nonetheless able to synchronize to a metronome and display normal sensitivity in terms of rhythmic pattern discrimination. The auditory perception–action dissociation that we found here and in our previous experiment is novel in that it involves normal (i.e., non-beat-deaf) participants, though unlike the time-series-based synchronization data used to study beat deafness, our study involves single-value judgments obtained after each stimulus has been presented. It would be interesting to see the extent to which the TAE arises in the tempo perception of beat-deaf individuals.

Studies of perception–action dissociation in vision have made use of various optical illusions, such as grasping objects that are part of a Müller–Lyer line or Ebbinghaus illusion, or examining eye saccades in an induced Roelofs effect, and they found that although perceptual reports were nonveridical, actions were resistant to the visual illusion. To be sure, these dissociations have been highly contested (e.g., Dassonville & Bala, 2004; Foster, Kleinholdermann, Leifheit, & Franz, 2012; Franz & Gegenfurtner, 2008). We are sensitive to the criticisms in these discussions, especially as our tapping task is a closed loop task, one whose error correction mechanisms have been well studied (see Repp, 2005; Repp & Su, 2013), while our tempo-judgment task is essentially an open loop between stimulus input and perceptual report (output). However, given the large and growing body of both behavioral and neurological evidence that links rhythm perception and production cited in the introduction (Maes et al., 2014; Ross et al., 2016), as well as various integrative models for rhythm perception and production (Fischinger, 2011; Kotz, Brown, & Schwartze, 2016; Vuust & Witek, 2014; see also Herwig, 2015), our initial hypothesis presupposed that moving along to the beat would provide a “kinematic input” for the mental representation that was being developed as participants listened to and tapped along with each stimulus. As this representation includes tempo information, as given by awareness of the beat rate, it follows that representations that involved tapping should reduce or eliminate the TAE. This does not seem to have happened. Rather, our participants’ low-level perception and reproduction of the beat rate (i.e., the appropriate tapping period) seems to have remained independent of their higher level cognitive appraisals of musical tempo, suggesting that higher-versus lower level temporal perceptions and actions may rely on different mechanisms, even in contexts where their spheres of attention and action overlap. Although clearly there is an auditory–motor linkage involved in rhythm perception and production—for without it, one could not synchronize a motor action to an auditory stimulus—it is not clear that motor simulations and/or common coding are involved in higher level cognitive appraisals of musical tempo. This reminds us of the differences that have been observed between phase-error versus period-error correction mechanisms in sensorimotor synchronization studies, the former, “being largely automatic and operating via phase resetting, and the other being mostly under cognitive control and, presumably, operating via a modulation of the period of an internal timekeeper” (Repp, 2005, p. 987).

Kivy (1993) characterized music as the *Fine Art of Repetition* and Margulis (2014) draws attention to the pervasiveness of repetition both within and between pieces of music. Margulis notes that repetition of auditory stimuli is useful for (a) learning, as repetitions are a form of rehearsal and memory reinforcement; (b) segmentation of the units of an auditory stream, as the onset of a repetition creates a clear articulation; and (c) generating expectations, as a current presentation of a previously heard auditory sequence generates particular expectations for its continuation and completion (Margulis, 2014, pp. 23–25). Our exploration of the TAE suggests another reason for the pervasiveness of repetition. Beyond our expectations for continuation and completion, our memories of music we have heard before may serve more broadly as frames of reference for our perception of tempo as well as other aspects (e.g., phrasing, expressive timing, intonation, timbre, dynamics). Teufel, Dakin, and Fletcher (2018) reported that, for vision, “high-level object representations interact with and sharpen early feature-detectors, optimising their performance for the current perceptual context” (Teufel, Dakin, and Fletcher, 2018, p. 1; see also Kok, Jehee, & de Lange, 2012, on visual expectation and perceptual sharpening, as well as Chennu et al., 2013, on auditory expectation and attention). The TAE and its “distortion” of tempo judgments may be an analogous form of perceptual sharpening, as it is manifest as “overstating” the relative differences among time-stretched musical stimuli. The TAE thus may indicate one mechanism by which our previous experiences of particular pieces of music may give us heightened sensitivities to subtle differences among performances.

Future research on the TAE should focus on improving our understanding of both tempo perception in the context of time-stretched musical stimuli and the effect of self-movement on rhythm perception. Regarding the former, it is still an open question whether the TAE represents a bona-fide temporal illusion, or simply a context-driven shift in decision criteria that emerges when time-stretched and original versions of a musical stimulus are related to a subrange within an absolute rating scale. Additional studies of the TAE that vary the experimental design by using different rating scales, a different experimental task (e.g., a standard-comparison task) and different ranges and subranges of stimulus tempos (both overlapping and nonoverlapping) may help clarify these questions. The TAE may also be related to the particular stimuli used in our original experiment, and their limitations. In using real-world musical recordings, one is limited in the extent to which time-stretching algorithms can be applied, as speeding up/slowing down a recording more than 7%–10% introduces audible processing artifacts. Using so-called artificial musical stimuli (whether composed for the experiment or based upon real compositions) rendered via a computer-controlled MIDI instrument would allow for the “same” stimulus to be presented over a wider range of tempos. Likewise, using stimuli from a wider range of musical styles, including purely percussive stimuli which lack pitch information, may shed additional light on the cues for the TAE. Regarding the effect of movement, greater precision in gathering data on participants’ self-motion, using motion capture or other systems, may give a clearer sense of the extent of bodily motion and the extent of its influence, as greater bodily motion (especially full body motion) would give rise to increased vestibular input (Phillips-Silver & Trainor, 2007, 2008). Lastly, more nuanced responses, which probe not only speed but also the character of the motion in terms of its continuity (smooth vs. halting), effort (easy vs. difficult), dynamics (high vs. low), and affective character may show a clearer relationship between acoustic cues, listener self-motion, and rhythmic perception.

More broadly, our findings suggest that the relationship between rhythm perception, memory, and bodily movement is more complex than previously observed. Judgments of musical tempo are made relative to multiple, nested frames of reference. These include acoustic factors beyond BPM such as loudness, register, surface event density, and spectral flux (Boltz, 2011; Drake et al., 1999; Eitan & Granot, 2009; Elowsson & Friberg, 2013). They also include contextual factors, including the tempos and movement characteristics as manifested in note-to-note patterns of timing and dynamics that are normative for a particular piece, style, or genre, tempered by each participants’ familiarity with these factors. Our results here suggest that in addition to those factors, a listener’s memories of different versions of the same (or perhaps similar) music can influence their perception of a temporally varied presentation.

## Footnotes

1 While latency issues with computer keyboards are well known (Fatin, 2015; Miron, Gouyon, Davies, & Holzapfel, 2013), given that the aim of having participants tap was to induce a focused motor engagement with the stimuli and to assess whether or not they were tapping at a given stimulus’s BPM rate (or integer multiple thereof), a small constant latency in our hardware and software was not a concern, given that these latencies are part and parcel of most instrument mechanisms, both acoustical and electronic (Mäki-Patola, 2005). To ensure that latency variability was not a factor in our measurements, we benchmarked the latency variability in the following way. While running the Max patch used in the experiment (using one of the MacBooks used in the experiment), we tapped on the spacebar with a hard plastic cylinder, which produced a sharp click at the moment of contact. An audio recording was made of those tapping sounds. The Max patch used for stimulus presentation and data collection produces a running time log of the taps, from which the intertap intervals (ITIs) are computed. A similar series of ITIs was derived from the audio recordings, using the initial sound onset as the temporal reference. In both the Max patch and the audio files, temporal resolution was 1 ms. The ITIs from both sources were aligned and compared. The average signed differences between the two time series was less than 1 ms, and the unsigned difference (absolute value) was less than 3 ms. This indicates both (a) that the latency, which we estimate to have been in the 10–15ms range (see Fatin, 2015), was quite stable, and (b) the variability was quite small—well below the 40–60-ms standard deviations we found in our participants tapping data (see Table 2).
